# Cigarette Smoke Increases Endothelial CXCL16-Leukocyte CXCR6 Adhesion *In Vitro* and *In Vivo*. Potential Consequences in Chronic Obstructive Pulmonary Disease

**DOI:** 10.3389/fimmu.2017.01766

**Published:** 2017-12-13

**Authors:** Patrice Marques, Aida Collado, Paula Escudero, Cristina Rius, Cruz González, Emilio Servera, Laura Piqueras, Maria-Jesus Sanz

**Affiliations:** ^1^Department of Pharmacology, Faculty of Medicine, University of Valencia, Valencia, Spain; ^2^Institute of Health Research INCLIVA, University Clinic Hospital of Valencia, Valencia, Spain; ^3^Neumology Unit, University Clinic Hospital of Valencia, Valencia, Spain

**Keywords:** leukocyte recruitment, chemokines, endothelial dysfunction, cardiovascular comorbidity, arterial endothelium, cigarette smoke, chronic obstructive pulmonary disease

## Abstract

Cardiovascular disease (CVD) is a major comorbidity in chronic obstructive pulmonary disease (COPD). Although the mechanism of its development remains largely unknown, it appears to be associated with cigarette consumption and reduced lung function. Therefore, the aim of this study was to investigate the potential link between water-soluble cigarette smoke extract (CSE)-induced endothelial dysfunction and the function of CXCL16/CXCR6 axis on the initial attachment of leukocytes, in addition to its possible impact on COPD-associated systemic inflammation. To do this, we employed several experimental approaches, including RNA silencing and flow cytometry analysis, the dynamic flow chamber technique, and intravital microscopy in the cremasteric arterioles of animals exposed to cigarette smoke (CS). CSE-induced arterial CXCL16 expression, leading to increased platelet–leukocyte and mononuclear cell adhesiveness. CSE-induced CXCL16 expression was dependent on Nox5 expression and subsequent RhoA/p38 MAPK/NF-κB activation. Flow cytometry analysis revealed that COPD patients (*n* = 35) presented greater numbers of activated circulating platelets (PAC-1^+^ and P-selectin^+^) expressing CXCL16 and CXCR6 as compared with age-matched controls (*n* = 17), with a higher number of CXCR6^+^-platelets in the smoking COPD group than in ex-smokers. This correlated with enhanced circulating CXCR6^+^-platelet–leukocyte aggregates in COPD patients. The increase in circulating numbers of CXCR6-expressing platelets and mononuclear cells resulted in enhanced platelet–leukocyte and leukocyte adhesiveness to CSE-stimulated arterial endothelium, which was greater than that found in age-matched controls and was partly dependent on endothelial CXCL16 upregulation. Furthermore, CS exposure provoked CXCL16-dependent leukocyte–arteriolar adhesion in cremasteric arterioles, which was significantly reduced in animals with a nonfunctional CXCR6 receptor. In conclusion, we provide the first evidence that increased numbers of CXCR6-expressing circulating platelets and mononuclear leukocytes from patients with COPD might be a marker of systemic inflammation with potential consequences in CVD development. Accordingly, CXCL16/CXCR6 axis blockade might constitute a new therapeutic approach for decreasing the risk of CVD in COPD patients.

## Introduction

Chronic obstructive pulmonary disease (COPD) is a complex respiratory disorder characterized by the progressive and largely irreversible decrement in lung function associated with an abnormal chronic inflammatory response of the lungs to noxious particles and gases, mostly from cigarette smoke (CS). COPD is considered a multisystem disease characterized by both pulmonary and systemic inflammation (1–3). The latter is believed to be responsible for many COPD comorbidities (1, 3, 4), one of the most relevant of which is cardiovascular disease (CVD) (1, 3, 4). Indeed, epidemiological studies show that smoking is a significant risk factor for heart disease including aneurysm formation and rupture, and stroke through atherosclerosis development (5). Endothelial dysfunction is an early event in atherogenesis, which leads to a pro-inflammatory and prothrombotic phenotype (6) and triggers the adhesion and subsequent migration of leukocytes. Accordingly, vascular dysfunction in smokers has been widely described (7).

Adhesive interactions between leukocytes and arterial endothelium precede leukocyte infiltration to the subendothelial space (6, 8). Chemoattractant molecules including chemokines can potentially recruit specific cell types and participate in the regulation of leukocyte trafficking (9). CXCL16 is a transmembrane chemokine expressed on endothelial, epithelial, and smooth muscle cells, as well as on macrophages, dendritic cells, B and T cells, and platelets (10, 11). CXCL16 was identified in human carotid endarterectomy samples and in lesions of apoE*^−/−^* mice fed a Western diet, pointing to a role in atherogenesis (12). CXCL16 is expressed in two distinct forms. Membrane-bound CXCL16 promotes the firm adhesion of cells expressing its cognate receptor, CXCR6. Proteolytic cleavage of membrane-bound CXCL16 releases soluble CXCL16, which acts as a chemoattractant for CXCR6^+^ cells (11).

The mechanisms by which CS promotes a pro-inflammatory and prothrombotic environment in the vessel wall remain largely unknown. We hypothesized that CS induces functional CXCL16 expression in arterial endothelium. Because CXCL16/CXCR6 axis is a potential candidate for CVD prediction and a target for therapeutic intervention (13), we also examined the levels of circulating CXCR6-expressing platelets and different leukocyte subsets in patients with COPD, and evaluated the implication for leukocyte adhesion to the dysfunctional arterial endothelium, a key event preceding atherogenesis.

## Materials and Methods

### Human *In Vitro* and *Ex Vivo* Studies

#### Cell Culture

Human umbilical arterial endothelial cells (HUAEC) were isolated by collagenase treatment (14) and maintained in human endothelial cell-specific medium (EBM-2) supplemented with endothelial growth medium (EGM-2) and 10% FBS. Cells up to passage 1 were grown to confluence to preserve endothelial features. Cells were incubated 16 h in medium containing 1% FBS prior to every experiment. Our previous studies have shown that human umbilical vein endothelial cells (HUVEC) do not behave like HUAEC in response to relevant cardiovascular stimuli such as angiotensin II (Ang-II) (15, 16). In this context, HUAEC and HUVEC showed dissimilar neutrophil and mononuclear cell adhesion when the same stimulus was applied.

#### Cigarette Smoke Extract (CSE) Preparation

Cigarettes were obtained from the Kentucky Tobacco Research and Development Center at the University of Kentucky. The composition of 3R4F research grade cigarettes is as follows: total particulate matter, 10.9 mg/cigarette; tar, 9.5 mg/cigarette; and nicotine, 0.726 mg/cigarette. A 10% CSE was prepared by bubbling smoke from one 3R4F cigarette into 10 ml of EGM-2 medium without FBS at a rate of one cigarette/2 min as previously described (17, 18). The pH of the CSE was adjusted to 7.4 and sterile filtered through a 0.22-µm filter. CSE preparation was standardized by measuring the absorbance (optical density = 0.86 ± 0.05) at a wavelength of 320 nm. The pattern of absorbance (spectrogram) observed at 320 nm showed very little variation between different preparations of CSE. CSE was freshly prepared for each experiment and diluted with culture medium supplemented with 0.1% FBS immediately before use. Control medium was prepared by bubbling air through 10 ml of culture medium without FBS, the pH was adjusted to 7.4, and the medium was filtered as described above. In preliminary studies, a range of concentrations of CSE were tested (0.1–3%) and a final concentration of 1% was used in all experiments since it was not cytotoxic to endothelial cells (18). This CSE concentration approximately corresponds to the exposure associated with smoking 1.5 packs per day as previously estimated (19), and is consistent with the amount smoked by the COPD patients in this study.

#### Quantitative RT-PCR

Human umbilical arterial endothelial cells were grown to confluence and stimulated with 1% CSE, INF-γ, or TNF-α (20 ng/ml) for 1 or 4 h. Reverse transcription was performed on 300 ng of total RNA with the TaqMan reverse transcription reagent kit. cDNA was amplified with specific primers for CXCL16 and GAPDH (all pre-designed by Applied Biosystems, Carlsbad, CA, USA) in a 7900HT Fast Real-Time PCR System (Applied Biosystem) using Universal Master Mix (Applied Biosystems). Relative quantification of the different transcripts was determined with the 2*^−^*^ΔΔCt^ method using GAPDH as endogenous control and normalized to control group.

#### Flow Cytometry

To assess CXCL16 endothelial expression, HUAEC were grown to confluence and stimulated with 1% CSE, INF-γ, or TNF-α (20 ng/ml) for 24 h. Then, endothelial cells were detached from culture flasks by scraping in ice-cold phosphate buffered saline (PBS) containing 0.05% NaN_3_ and 0.2% BSA and recovered by centrifugation. HUAEC were washed and incubated at 2 × 10^6^ cells/ml with an APC-conjugated monoclonal antibody against human CXCL16 (1 µg/ml) in PBS with 0.2% BSA and 0.05% NaN_3_ for 1 h on ice. After two washes, cells were suspended in PBS containing 2% paraformaldehyde. The fluorescence signal of the labeled cells was then analyzed by flow cytometry. The expression of CXCL16 (APC-fluorescence) was expressed as the mean of fluorescence intensity.

To determine the grade of platelet activation, PAC-1^+^ platelets (detects activated integrin α_IIb_β_3_) and the expression of P-selectin (CD62P) were measured in platelets from active or not active smokers COPD patients and control-matched individuals by flow cytometry. Duplicate samples (25 µl) of citrated blood, diluted 1/10 in glucose buffer (1 mg/ml glucose in PBS/0.35% BSA), were incubated in the dark for 30 min with a CF-conjugated monoclonal antibody against human CD41 (5 µl) and an FITC-conjugated monoclonal antibody against human PAC-1 (2.5 µl) or with an APC-conjugated monoclonal antibody against human P-selectin (5 µl). Samples were run in a flow cytometer (FACSVerse flow cytometer, BD Biosciences, Franklin Lakes, NJ, USA). CD41^+^ (platelets) populations were selected according to the gating strategy illustrated in Figure SI in Supplementary Material and expressed as the percentage of positive platelets.

To determine the expression of CXCR6 and CXCL16 on platelets and CXCR6 on circulating neutrophils, monocytes, and lymphocytes from active or not active smokers COPD patients and control-matched individuals, duplicate samples (100 µl) of heparinized whole blood were incubated in the dark for 30 min with saturated amounts (10 µl) of PE-conjugated monoclonal antibodies against human CXCR6 or CXCL16. In some experiments, blood samples were incubated with EDTA (10 mM, for 15 min at 37°C) to promote platelet dissociation as described (20). Red blood cells were lysed and leukocytes were fixed using a commercial lysis buffer. Samples were run in a flow cytometer and the expression of CXCR6 (PE fluorescence) was measured on CD41^+^ (platelets), CD16^+^CD14*^−^* (neutrophils), CD14^+^ (monocytes), and CD3^+^ (lymphocytes) populations according to the gating strategy illustrated in Figures SII–SIV in Supplementary Material.

#### Leukocyte–Endothelial Cell Interactions under Flow Conditions

A dynamic flow chamber assay was performed using human neutrophils and mononuclear cells obtained from buffy coats of healthy donors by Ficoll Hypaque density gradient centrifugation (14). HUAEC were grown to confluence and stimulated with 1% CSE or TNF-α (20 ng/ml) for 24 h. The flow chamber (GlycoTech, Rockville, MD, USA) was assembled and placed onto an inverted microscope stage. Then, freshly isolated neutrophils and mononuclear cells (1 × 10^6^/ml) were perfused across the endothelial monolayers and leukocyte–endothelial cell interactions were determined. In all experiments, leukocyte interactions were determined after 5 min perfusion at 0.5 dyn/cm^2^. Cells interacting on the surface of the endothelium were visualized and recorded (20× objective, 10× eyepiece) using phase-contrast microscopy (Axio Observer A1 Carl Zeiss microscope, Thornwood, NY, USA).

In parallel, some plates were incubated with a monoclonal neutralizing antibody against human CXCL16 (2 µg/ml) or with an isotype-matched control antibody (MOPC-21, 2 µg/ml) 10 min before blood perfusion. To evaluate the contribution of platelets to leukocyte adhesion, the experiments were carried out in heparinized blood incubated or not with EDTA (10 mM, for 15 min, 37°C).

For human studies, diluted whole blood (1/10 in Hanks balanced salt solution) from COPD patients and age-matched controls was perfused across unstimulated or 1% CSE-stimulated endothelial monolayers and leukocyte–endothelial cell interactions were determined. Similarly, in some experiments, plates were incubated with a monoclonal neutralizing antibody against human CXCL16 (2 µg/ml) or with a control antibody (MOPC-21, 2 µg/ml) 10 min before blood perfusion. Again, the experiments were carried out in heparinized blood incubated or not with EDTA (10 mM, for 15 min, 37°C) to determine platelet contribution to leukocyte adhesion.

#### Immunofluorescence

Confluent endothelial cells were grown on glass coverslips and stimulated with 1% CSE, INF-γ, or TNF-α (20 ng/ml) for 24 h. The cells were fixed with 4% paraformaldehyde and blocked in a PBS solution containing 1% BSA. Then, cells were incubated at 4°C overnight with a primary goat monoclonal antibody against human CXCL16 (1:200 dilution) in a 0.1% BSA/PBS solution, followed by incubation with a secondary FITC-conjugated rabbit anti-goat monoclonal antibody (1/1,000 dilution) at room temperature for 45 min. Cell nuclei were counterstained with 4′-6-diamidino-2-phenylindole (DAPI). Images were captured with a fluorescence microscope (Axio Observer A1, Carl Zeiss, NY, USA) equipped with a 40× objective lens and a 10× eyepiece.

#### Transfection of Nox2, Nox4, Nox5, or RhoA siRNA

Transfection was carried out with Lipofectamine RNAiMAX following the manufacturer’s instructions. To determine silencing efficiency, Nox2, Nox4, Nox5, and RhoA expression was determined by western blotting of cell lysates at 48 h post-transfection.

#### Western Blotting

After treatments, cells were washed, detached, collected, and centrifuged at 15,000 *g* at 4°C for 30 min to yield the whole extract. Protein content was determined by the Bradford method. Samples were denatured, subjected to SDS-PAGE using a 10% running gel, and transferred to nitrocellulose membrane. Non-specific binding sites were blocked with 3% BSA in TBS solution, and membranes were incubated overnight with a mouse polyclonal antibody against human Nox2 (0.2 µg/ml), a rabbit polyclonal antibody against human Nox4 (2 µg/ml), a rabbit polyclonal antibody against human Nox5 (2 µg/ml), or a mouse polyclonal antibody against human RhoA (5 µg/ml). Subsequently, membranes were washed and further incubated for 1 h with the corresponding secondary HRP-linked antibody; anti-rabbit IgG (1:2,000 dilution), anti-goat IgG (1:2,000 dilution), or anti-mouse IgG (1:2,000 dilution) and developed using the ECL system. Signals were recorded with a luminescent analyzer (FujiFilm image Reader LAS1000, Fuji, Tokyo, Japan) and analyzed using ImageJ (Windows free version).

#### Experimental Protocols

To evaluate the potential involvement of NADPH and xanthine oxidase (XO) on CSE-induced responses, cells were incubated for 1 h with an NADPH oxidase inhibitor (apocynin, 30 µM) or with a XO inhibitor (allopurinol, 100 µM) and then stimulated with 1% CSE for 24 h. The doses of these compounds were used as previously described (18, 21). Because the NADPH oxidase isoforms Nox2, Nox4, and Nox5 are all expressed in endothelial cells (22, 23), in subsequent experiments HUAEC were transfected with either control or Nox2, Nox4, Nox5-specific siRNAs as described above. HUAEC were stimulated with 1% CSE 48 h post-transfection and CXCL16 expression was evaluated by western blot.

To investigate the possible contribution of RhoA to CSE-induced CXCL16 expression, HUAEC were transfected for 48 h with control or RhoA-specific siRNA prior to CSE stimulation, and CSE-induced responses were measured 24 h later by flow cytometry.

In another set of experiments, cells were treated with a RhoA inhibitor (C3 transferase, 2 µg/ml) 4 h before CSE stimulation and CXCL16 expression was measured at 24 h by flow cytometry.

Finally, to further dissect the signaling pathways involved in CS-induced responses, endothelial cells were pretreated with inhibitors of ERK1/2 (PD098059, 20 µM), p38-MAPK (SB202190, 20 µM), and NF-κB (MOL-294, 2.5 µM) 1 h prior to Ang-II stimulation. These concentrations have been employed in previous studies to successfully inhibit ERK1/2, p38-MAPK, or NF-κB activation (24, 25). CXCL16 expression was determined by flow cytometry 24 h after stimulation with 1% CSE.

#### Human Study Populations

All investigations with human samples conformed to the principles outlined in the Declaration of Helsinki and were approved by the institutional ethics committee at the University Clinic Hospital of Valencia. Written informed consent was obtained from all subjects.

A total of 52 subjects (35 COPD patients and 17 age-matched control subjects without COPD) were included in the study. COPD patients and control subjects were recruited by the Pneumology Unit at University Clinic Hospital of Valencia, Valencia, Spain. All patients had COPD confirmed by medical history, clinical and functional examinations, according to criteria established by the American Thoracic Society [Standards of diagnosis and care of patients with chronic obstructive pulmonary disease. *Am J Respir Crit Care Med* (1995) 152(Suppl):77–120].

The inclusion criteria for patients entering in the study were as follows: >40 years of age, smoking history of 10 packs/year, COPD confirmed by the post-bronchodilator ratio of low forced expiratory volume in 1 s (FEV 1) and forced vital capacity (FVC), FEV 1/FVC ratio <0.70 and the post-bronchodilator FEV 1 <80%, clinically stable with no exacerbations in the 8 weeks prior to the study. One pack-year was defined as smoking 20 cigarettes per day for 1 year. Exclusion criteria were the following: major vascular events (coronary artery disease, peripheral arterial disease, or stroke), asthma, autoimmune diseases, infection or inflammatory disease (including personal history of allergy), or the use of drugs capable of modifying inflammation that cannot be withdrawn 8 weeks before starting the study, alcohol consumption >30 g per day, and smoking history of <10 packs/year. Individuals in the control group were volunteers seen at the respiratory function laboratory for routine preoperative assessment. Their age was >40 years, they were non smokers, had no history of pulmonary disease or respiratory symptoms, had a normal spirometry and conformed with most of the exclusion criteria described for COPD patients. Spirometry was performed on a Master Scope (Jaeger, Germany) after inhalation of 0.4 mg salbutamol. A minimum of three airflow and volume tracings were obtained and the highest value for FEV 1 and FVC as percent-predicted normal were used for calculations. Most of the patients included in the study presented moderate COPD according to the GOLD classification [Global Initiative for Chronic Obstructive Lung Disease. Global strategy for the diagnosis, management, and prevention of COPD (January 2015), available from http://www.goldcopd.org]. Accordingly, 11.8% of the smokers with COPD were GOLD1 (mild), 58.8% were GOLD2 (moderate), and 29.4% were GOLD3 (severe). In the ex-smokers COPD patient group, 5.6% were GOLD1 (mild), 38.9% were GOLD2 (moderate), 44.4% were GOLD3 (severe), and 11.1% were GOLD4 (very severe). Those active smokers COPD patients had a cigarette within 2 h prior to lung function testing and blood sample collection. Clinical features of patients and age-matched controls are shown in Table SI in Supplementary Material. Given that COPD patients usually present additional comorbidities, the comorbidities of the three groups under investigation have been depicted in Table SII in Supplementary Material.

#### Soluble CXCL16 Quantification

Heparinized human whole blood (10 U heparin/ml) from COPD patients and aged-matched volunteers was collected. Before centrifugation to obtain plasma, additional heparin was added to the blood sample (to 100 U/ml). This procedure was used to help dissociate chemokines from blood cells. Plasma samples were stored at −80°C. Human CXCL16 was measured in plasma by ELISA. Results are expressed as picomolar chemokine in plasma.

### Animal Studies

The animal protocol conforms to the Guide for the Care and Use of Laboratory Animals published by the US National Institutes of Health (NIH publication No. 85-23, revised 1996) and was approved by the Ethics Review Board of the University of Valencia.

Male C57BL/6 mice (22–30 g weight) carrying a targeted knock-in of GFP to disrupt the CXCR6 gene were used. Male heterozygous CXCR6^gfp/+^ mice were used as controls (CXCR6^−/+^) and homozygous CXCR6^gfp/gfp^ animals that do not express functional CXCR6 receptor were used as CXCR6-deficient mice (CXCR6^−/−^).

Animal colonies were bred and maintained under specific pathogen-free conditions. Mice were fed with autoclaved balanced diet and water.

#### CS Exposure

Exposure of mice to CS was carried out using a modified method previously described (18, 26). In brief, mice were placed in a plexiglass chamber (volume of 20 l) covered by a disposable filter. The smoke produced by cigarette burning was introduced at a rate of 25 ml/min into the chamber with a continuous airflow generated by a mechanical ventilator, with no influence on the chamber temperature (<0.1°C variation). The animals received smoke from five 3R4F research-grade cigarettes. Exposure lasted approximately 35 min: 5 min in the smoke-air condition and 1 min without smoke. They were subjected to two exposures per day with a 60-min smoke-free interval during 3 days. Experiments were carried out 16 h after the last exposure.

#### Intravital Microscopy

The mouse cremaster preparation used in this study was similar to that described previously (27). Mice were anesthetized by i.p. injection with a mixture of xylazine hydrochloride (10 mg/kg) and ketamine hydrochloride (200 mg/kg). Additional anesthetic (30 µl, i.v.) was administered as required to maintain profound anesthesia. A polyethylene catheter was placed in the jugular vein to permit the intravenous administration of additional anesthetic. The cremaster muscle was dissected free of tissues and exteriorized onto an optical clear viewing pedestal. The muscle was cut longitudinally with a cautery and held flat against the pedestal by attaching silk sutures to the corners of the tissue. The muscle was then perfused continuously at a rate of 1 ml/min with warmed bicarbonate-buffered saline (pH 7.4).

The cremasteric microcirculation was observed using an intravital microscope (Nikon Optiphot-2, SMZ1, Badhoevedorp, The Netherlands) equipped with a 50× objective lens (Nikon SLDW, Badhoevedorp, The Netherlands) and a 10× eyepiece. A video camera (Sony SSC-C350P, Koeln, Germany) mounted on the microscope projected the image onto a color monitor and the images were CCD recorded for playback analysis. Cremasteric arterioles (20–40 µm in diameter) were selected for study. Vessel diameter was measured online by using a video caliper (Microcirculation Research Institute, Texas A&M University, College Station, TX, USA).

The number of adherent leukocytes was determined offline during playback of the recorded images. A leukocyte was defined as adherent to arteriolar endothelium if it was stationary for at least 30 s. Leukocyte adhesion was expressed as the number per 100 µm length of vessel per 5 min. Leukocyte responses were averaged in three to five randomly selected arterioles in each animal.

#### RT-PCR

Reverse transcription was performed on 300 ng of total RNA with TaqMan reverse transcription reagents kit. cDNA was amplified using standard protocols employing the following primers: mouse CXCL16 forward, 5′-GCT TTG GAC CCT TGT CTC TTG C-3′, reverse 5′-GTG CTG AGT GCT CTG ACT ATG TGC-3′; GAPDH forward: 5′-TGACCACAGTCCATGCCATC-3′ and reverse 5′-GACGGACACATTGGGGGTAG-3′; in a 7900HT Fast real-time PCR System (Applied Biosystem) using Universal Master Mix (Applied Biosystems). Relative quantification of the different transcripts was determined with the 2^−ΔΔCt^ method using GAPDH as endogenous control and normalized to control group.

#### Histology and Immunofluorescence

Immunofluorescence studies were carried out following a similar protocol to that previously described (28). Once intravital microscopy determinations were performed, mice were sacrificed and the cremaster muscle was isolated and fixed in 4% paraformaldehyde for 10 min. Muscles were incubated in 0.2% Triton X-100, 1% BSA, and 0.5% horse serum in PBS for 2 h. Then, muscles were incubated overnight at 4°C with a primary rabbit anti-mouse CXCL16 antibody (1/200 dilution) or PE-conjugated anti-mouse CD31 (PECAM-1) antibody (1/100 dilution). Samples were washed with PBS and incubated for 1.5 h at room temperature with Alexa Fluor 488-conjugated donkey anti-rabbit secondary antibody (1/500 dilution). All antibodies were diluted in 0.1% PBS/BSA. Muscles were then mounted with Slowfade Gold Reagent (Invitrogen, Eugene, OR, USA). Images were acquired with a fluorescence microscope (Axio Observer A1, Carl Zeiss, NY, USA) equipped with a 40× objective lens and a 10× eyepiece.

### Additional Materials

Endothelial basal medium-2 (EBM-2) supplemented with endothelial growth medium-2 (EGM-2) and FBS were purchased from Lonza Iberica (Barcelona, Spain). Ketamine and xylazine hydrochloride were from ORION Pharma (Espoo, Finland). Apocynine, allopurinol, SB202190, PD098059, hematoxylin, the mouse anti-human β-actin monoclonal antibody (clone AC-15), the monoclonal antibody IgG1 (MOPC-21), and the rabbit polyclonal anti-human Nox5 antibody were purchased from Sigma-Aldrich (Madrid, Spain). The APC-conjugated rat monoclonal anti-human CXCL16, the recombinant human CXCL16, the rat polyclonal anti-human CXCL16, and the biotinylated goat polyclonal anti-human CXCL16 antibodies were purchased from R&D Systems (Abingdon, UK). The rabbit polyclonal anti-human Nox4 and the mouse monoclonal anti-human RhoA antibodies were from Abcam (Cambridge, UK). The mouse monoclonal anti-human Nox2 (clone NL7) antibody was purchased from Serotec (Oxford, UK). Neutravidin-HRP was supplied by Perbio Science (Northumberland, UK). Ficoll-Paque TM plus and ECL developer were purchased from GE Healthcare (Chalfont St. Giles, UK). DAPI, TRIzol isolation reagent and the FITC-conjugated rabbit anti-goat secondary antibody were from Molecular Probes-Invitrogen (Carlsbad, CA, USA). The secondary antibodies, HRP-linked anti-goat and HRP-linked anti-rabbit were purchased from Dako (Glostrup, Denmark). The RhoA-specific siRNA and Ultra TMB-ELISA were purchased from Thermo Fisher Scientific Inc. (Kalamazoo, MI, USA). The FITC-conjugated monoclonal antibody against human PAC-1 and the lysing solution were from BD Biosciences (San Jose, CA, USA). The CF-conjugated mouse monoclonal antibody against human CD41, the APC-conjugated mouse monoclonal antibody against human P-selectin, and the rabbit mAb against mouse CXCL16 were from Immunostep (Salamanca, Spain). The cell-permeable C3 transferase (RhoA inhibitor) was from Cytoskeleton Inc. (Denver, CO, USA). Slowfade Gold Reagent and lipofectamine RNAiMAX were from Invitrogen (Eugene, OR, USA). Nox2-, Nox4-, and Nox5-specific siRNAs were purchased from Pharmacon (Lafayette, CO, USA). TaqMan reverse transcription reagents kit was from Applied Biosystems (Perkin-Elmer Corporation, Carlsbad, CA, USA). Maxima First Strand cDNA Synthesis kit and Luminar Color HiGreen HigBox qPCR Master Mix were from Fermentas, Thermo Fisher Scientific (Waltham, MA, USA). The PE-conjugated rat monoclonal anti-mouse CD31 antibody (clone 390) was from eBioscience (Hatfield, UK). MOL-294 was kindly donated by Dr. Kahn (Department of Pathobiology, University Washington, Seattle, WA, USA).

### Statistical Analysis

Values were expressed as mean ± SEM. For comparisons between two groups, Student’s *t*-test was used in data that passed normality (Kolmogorov*–*Smirnov test), otherwise, the non-parametric Mann–Whitney *U* test was performed. Data within multiple groups were compared by one-way analysis of variance including Newman–Keuls *post hoc* test for multiple comparisons. *P*-values less than 0.05 were considered to be significant.

## Results

### CSE Induces Functional CXCL16 Expression in HUAEC

**Figure 1:**
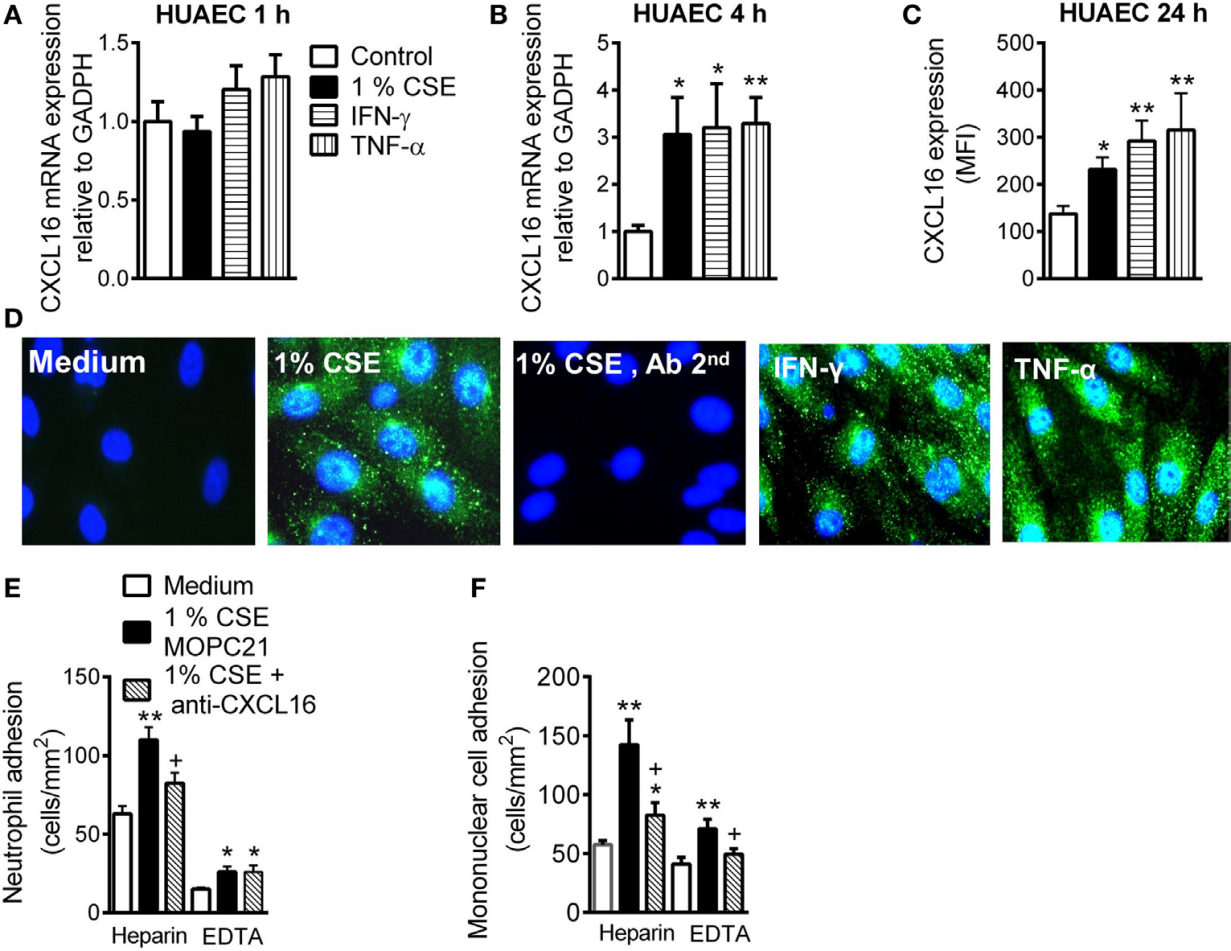
Expression and function of CXCL16 in cigarette smoke extract (CSE)-stimulated human umbilical arterial endothelial cells (HUAEC) and effect of CXCL16 neutralizing antibody on neutrophil and mononuclear leukocyte adhesion. HUAEC were stimulated with 1% CSE, INF-γ, or TNF-α (20 ng/ml) for 1, 4, or 24 h. Relative quantification of mRNA levels for CXCL16 and GAPDH after **(A)** 1 h and **(B)** 4 h (*n* = 5–8 independent experiments). Columns show fold increase in CXCL16 mRNA expression relative to control GAPDH. Values are expressed as mean ± SEM of the 2^−ΔΔCt^ values. *P < 0.05 or **P < 0.01 relative to values in the medium group. **(C)** Protein expression was determined by flow cytometry. Results are expressed as mean of fluorescence intensity (MFI) (*n* = 7–8 independent experiments). Values are expressed as mean ± SEM. *P < 0.05 or **P < 0.01 relative to values in the medium group. **(D)** Following a similar protocol, CXCL16 was visualized in non-permeabilized HUAEC by immunofluorescence (green). Nuclei were counterstained with 4′6-diamidino-2-phenylindole (DAPI) (*n* = 4–5 independent experiments). **(E,F)** Endothelial cells were stimulated with 1% CSE for 24 h. Some cells were incubated with a CXCL16 neutralizing antibody (2 µg/ml) or an irrelevant isotype-matched monoclonal antibody (MOPC-21, 2 µg/ml). Subsequently, human neutrophils **(E)** or mononuclear cells **(F)** (1 × 10^6^ cells/ml) incubated with or without EDTA were perfused over the monolayers for 5 min at 0.5 dyn/cm^2^ and leukocyte accumulation quantified (*n* = 5–7 independent experiments). Values are expressed as the mean ± SEM. *P < 0.05 or **P < 0.01 relative to values in the medium group; ^+^*P* < 0.05 relative to the stimulus MOPC-21-treated group.

Whereas no differences were found for the expression of CXCL16 mRNA in HUAEC after stimulation with CSE, INF-γ, or TNF-α for 1 h (Figure 1A), prolonged stimulation (4 h) led to a significant increase in CXCL16 expression (Figure 1B). Flow cytometry analysis of HUAEC after 24 h exposure to CSE, INF-γ, or TNF-α revealed a significant increase in protein levels of CXCL16 (Figure 1C), which was confirmed by immunocytochemistry (Figure 1D).

To investigate the functional role of CSE-induced endothelial CXCL16 expression, freshly isolated human neutrophils or mononuclear cells were perfused across HUAEC monolayers. When compared with unstimulated cells, a significant increase in neutrophil and mononuclear cell arrest was observed in CSE-stimulated HUAEC, although greater adhesion was observed when platelets were bound (heparin) than unbound (EDTA) to leukocytes (Figure 1E). Neutralization of CXCL16 activity on the endothelial cell surface resulted in a significant reduction in CSE-induced neutrophil/platelet adhesion to HUAEC by 60% (Figure 1E). By contrast, no neutralizing activity was detected in platelet-free neutrophils (Figure 1E). CXCL16 blockade resulted in a significant reduction of mononuclear cell/platelet (heparin) arrest to CSE-stimulated HUAEC (71% inhibition, Figure 1F). While mononuclear cell adhesion was still evident in CSE-stimulated HUAEC when platelets were dissociated from leukocytes (EDTA), impaired mononuclear leukocyte attachment was detected after CXCL16 HUAEC neutralization (72% inhibition, Figure 1F). Similarly, neutralization of endothelial CXCL16 activity caused a significant reduction in TNF-α-induced neutrophil/platelet adhesion to HUAEC, but not in the absence of bound platelets (Figure SIVA in Supplementary Material). Regarding mononuclear cell adhesion, CXCL16 blockade also provoked a significant reduction of mononuclear cell arrest to TNF-α-stimulated HUAEC whether they were bound or not to platelets (Figure SVB in Supplementary Material).

### Nox5 Gene Silencing Inhibits CSE-Induced Endothelial CXCL16 Expression

**Figure 2:**
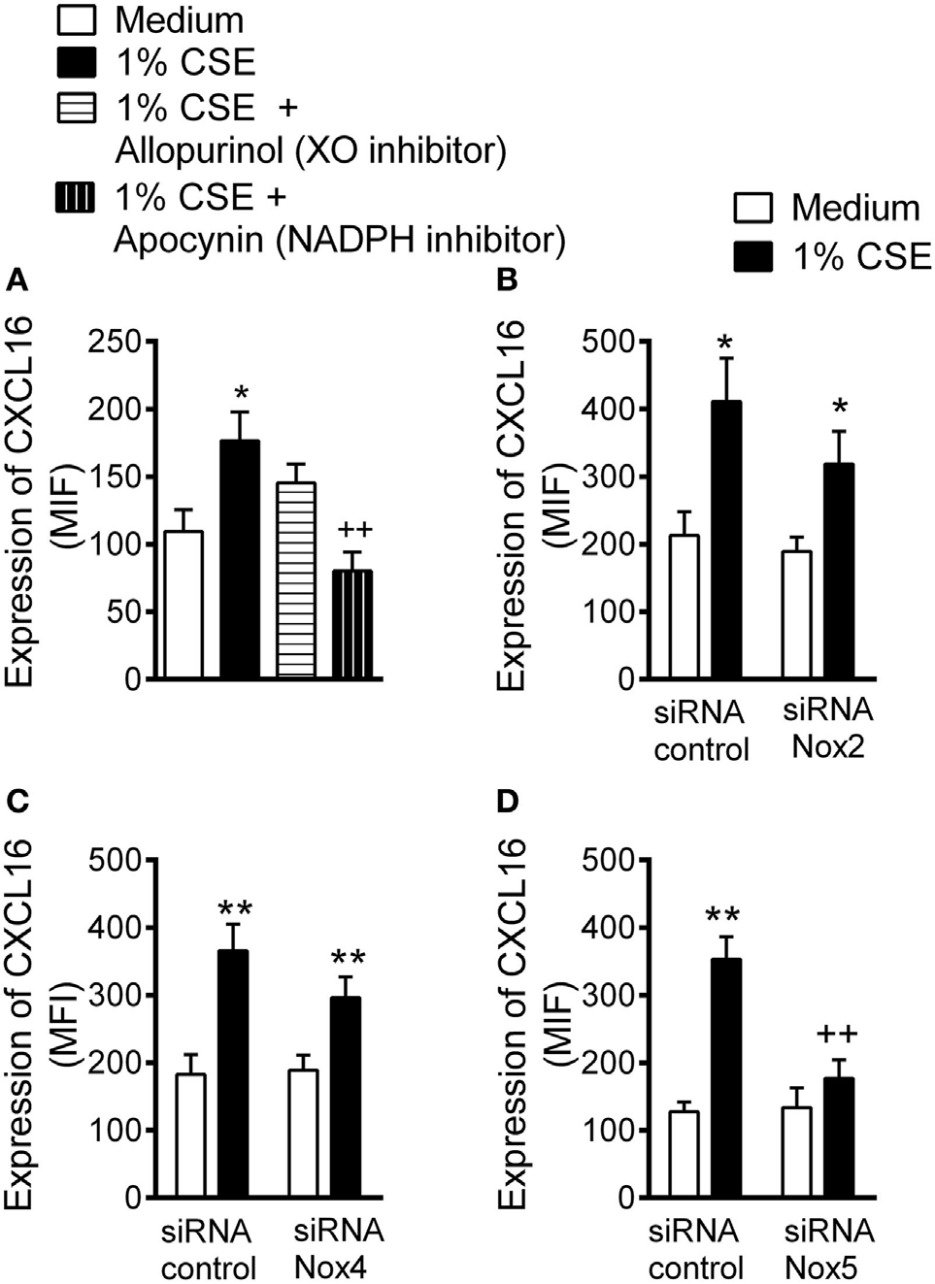
Inhibition of cigarette smoke extract (CSE)-induced CXCL16 expression in arterial endothelial cells. **(A)** CXCL16 expression was determined by flow cytometry in human umbilical arterial endothelial cells preincubated or not with apocynin (30 µM) or allopurinol (100 µM) for 1 h and then stimulated with 1% CSE for 24 h (*n* = 7 independent experiments). Results are expressed as mean of fluorescence intensity (MFI). Values are expressed as the mean ± SEM. *P < 0.05 relative to values in the medium group; ^++^*P* < 0.01 relative to 1% CSE group. Endothelial cells were transfected with **(B)** Nox2, **(C)** Nox4, or **(D)** Nox5 siRNA or control siRNA. At 48 h post-transfection, cells were stimulated with 1% CSE for 24 h. CXCL16 expression was determined by flow cytometry. Results are expressed as MFI (*n* = 5–11 independent experiments). Values are expressed as mean ± SEM. *P < 0.05 or ** *P* < 0.01 relative to values in the medium group; ^++^*P* < 0.01 relative to 1% CSE group in control siRNA transfected cells.

Water-soluble components of CS promote reactive oxygen species (ROS) generation (18, 29). Because NADPH oxidases (Nox) and XO are important vascular sources of ROS (18), we first demonstrated that non-specific inhibition of NADPH oxidases but not XO inhibition diminished CSE-induced CXCL16 expression (Figure 2A).

Endothelial cells mainly express the NADPH family members Nox2, Nox4, and Nox5 (22, 23). A significant reduction in the levels of Nox2, Nox4, and Nox5 protein was evident 48 h after treatment with their respective siRNA (Figure SVI in Supplementary Material). Notably, whereas Nox2 or Nox4 silencing had no significant impact on CSE-induced CXCL16 expression in HUAEC (Figures 2B,C), silencing of Nox5 resulted in an 81% reduction of CXCL16 expression in HUAEC (Figure 2D).

### RhoA, p38 MAPK, and NF-κB Activation Are Involved in CSE-Induced CXCL16 Expression in HUAEC

**Figure 3:**
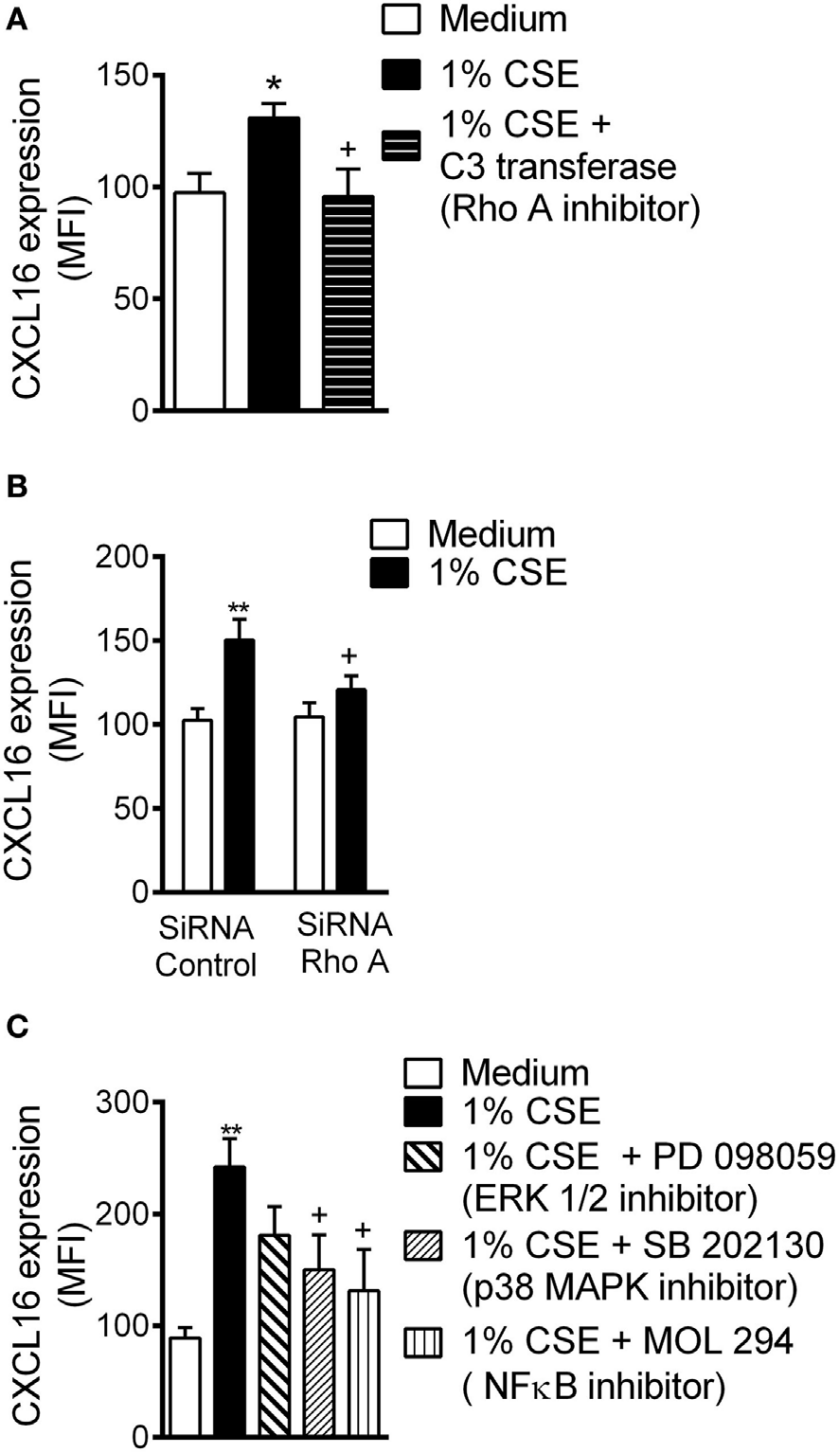
Cigarette smoke extract (CSE)-induced CXCL16 overexpression is decreased by RhoA, p38 mitogen-activated protein kinase (MAPK), and nuclear factor (NF)-κB inhibition in human umbilical arterial endothelial cells (HUAEC). **(A)** CXCL16 expression was determined by flow cytometey in endothelial cells preincubated or not with a RhoA inhibitor (C3 transferase, 2 µg/ml) for 4 h and then stimulated with 1% CSE for 24 h. Results are expressed as mean of fluorescence intensity (MFI) (*n* = 5 independent experiments). Values are expressed as mean ± SEM. *P < 0.05 relative to values in the medium group; ^+^*P* < 0.05 relative to 1% CSE group. **(B)** HUAEC were transfected with RhoA siRNA or control siRNA. At 48 h post-transfection, cells were stimulated with 1% CSE for 24 h. CXCL16 expression was determined by flow cytometry. Results are expressed as MFI (*n* = 7 independent experiments). Values are expressed as mean ± SEM. **P < 0.01 relative to values in the medium group; ^+^*P* < 0.05 relative to their respective group in control siRNA-transfected cells. **(C)** HUAEC were stimulated with 1% CSE for 24 h. Some cells were pretreated with PD098059 (20 µM), SB202130 (20 µM), or MOL-294 (2.5 µM) for 1 h before CSE stimulation. CXCL16 expression was determined by flow cytometry. Results are expressed as MFI (*n* = 4–7 independent experiments). Values are expressed as mean ± SEM. **P < 0.01 relative to values in the medium group; ^+^*P* < 0.05 relative to values in the 1% CSE group.

RhoA is activated by CS and participates in subsequent endothelial dysfunction (30). Inhibition of RhoA activity or depletion of RhoA protein by RNA interference led to a significant reduction in CXCL16 expression in CSE-treated HUAEC (Figures 3A,B, respectively).

Cigarette smoke can activate endothelial mitogen-activated protein kinase (MAPK) signaling cascades (18), which can modulate downstream targets such as NF-κB (31), leading to mononuclear cell recruitment. Pretreatment of arterial endothelial cells with a p38 MAPK inhibitor or with an NF-κB inhibitor, but not with an ERK1/2 inhibitor, significantly decreased surface expression of CXCL6 on CSE-stimulated cells (Figure 3C).

### Platelet Activation and Expression of CXCL16 and CXCR6 is Upregulated in Patients with COPD Active Smokers and Ex-Smokers

**Figure 4:**
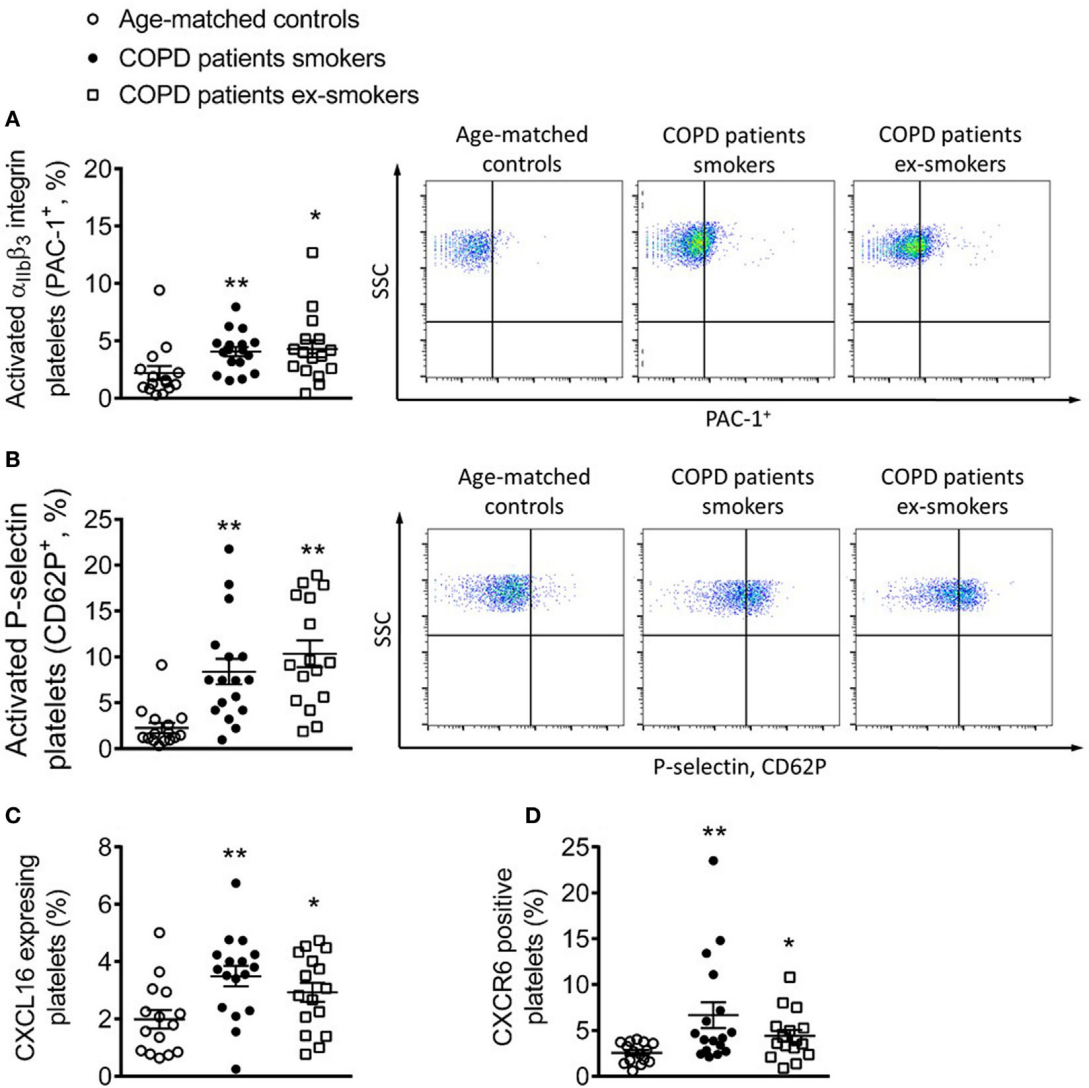
Percentage of circulating platelets expressing PAC-1, P-selectin, CXCL16, and CXCR6 from active and not active smoking chronic obstructive pulmonary disease (COPD) patients and aged-matched controls by flow cytometry. Platelets were stained with conjugated antibodies against **(A)** CD41 and PAC-1, **(B)** CD41 and P-selectin, **(C)** CD41 and CXCL16, and **(D)** CD41 and CXCR6. Results are expressed as percentage of positive cells (*n* = 15 aged-matched controls, *n* = 17 active smokers COPD patients, *n* = 16 ex-smokers COPD patients). Values are expressed as mean ± SEM. *P < 0.05 or **P < 0.01 relative to values in the control group.

A significant increase in the percentage of platelets expressing PAC-1 and P-selectin, which are platelet activation markers, was detected in patients with COPD when compared with control subjects, but no differences were encountered between active and ex-smokers COPD patients Figures 4A,B. Notably, the percentage of circulating platelets expressing CXCL16 and CXCR6 was significantly higher in patients with COPD than in control subjects (Figures 4C,D). Although the percentage of CXCR6^+^-platelets was higher in the smokers COPD group than in ex-smokers, this did not reach significance.

### CXCR6 Expression on Monocytes and Lymphocytes Is Upregulated in Patients with COPD

**Figure 5:**
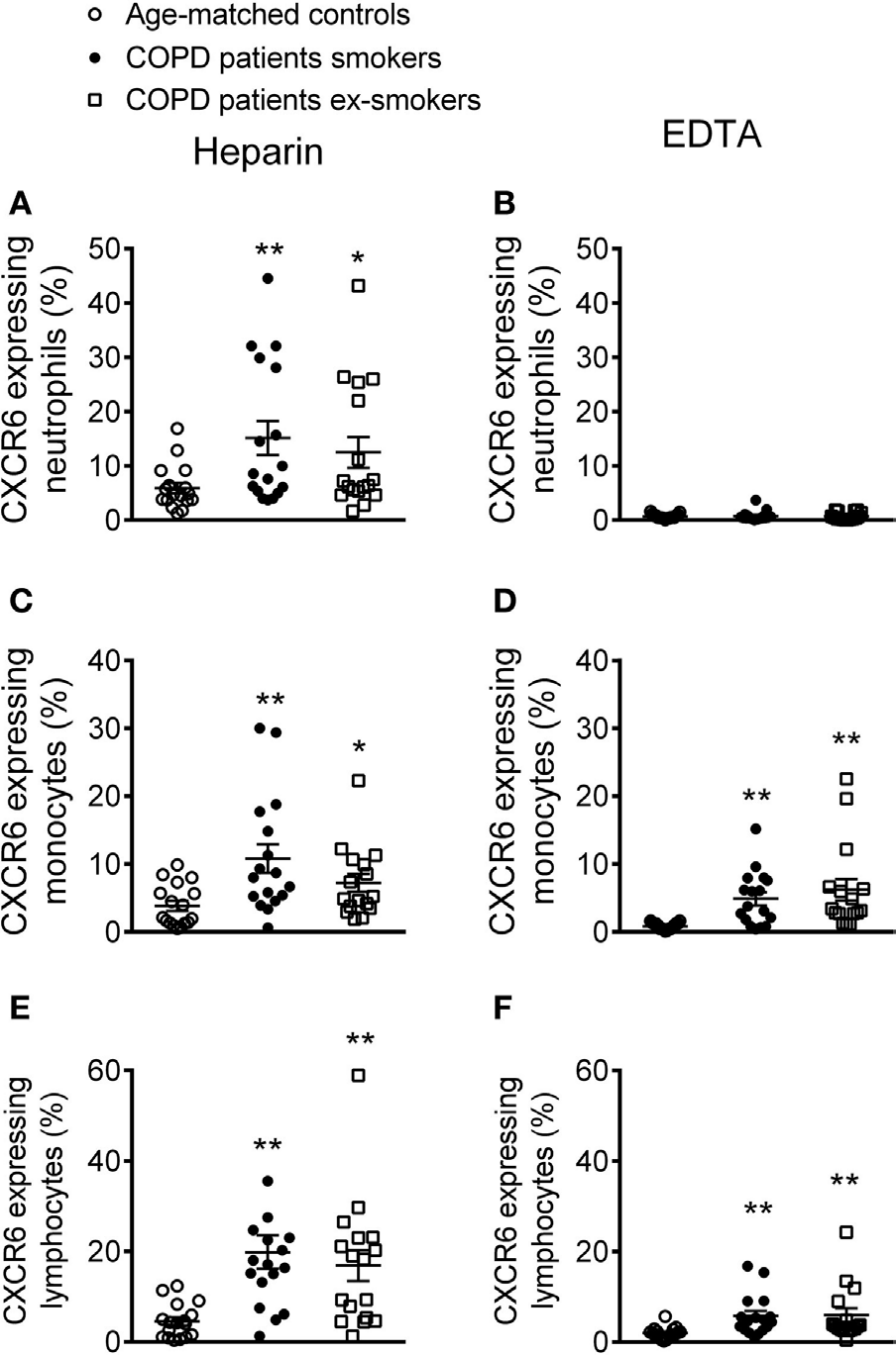
Percentage of CXCR6-expressing leukocytes in different circulating subpopulations from active and not active smoking chronic obstructive pulmonary disease (COPD) patients and aged-matched controls by flow cytometry. Heparinized whole blood was co-stained with specific markers for **(A,B)** neutrophils, **(C,D)** monocytes, and **(E,F)** lymphocytes and CXCR6. Blood samples were incubated or not with EDTA. Results are expressed as percentage of positive cells (*n* = 17 aged-matched controls, *n* = 17 active smokers COPD patients, *n* = 16 ex-smokers COPD patients). Values are expressed as mean ± SEM. *P < 0.05 or **P < 0.01 relative to values in the control group.

The number of circulating CXCR6-expressing neutrophils in heparinized whole blood was significantly higher in patients with COPD than in control subjects (Figure 5A). Although no significant differences in this number were found between active or ex-smokers in the COPD group, a tendency toward an increase in neutrophil numbers was found in the active smokers group (Figure 5A). This increase was abolished when platelets were dissociated from leukocytes (Figure 5B). CXCR6-expressing monocytes and lymphocytes were also greater in number in COPD patients than in age-matched controls (Figures 5C–F); while the number was greater when platelets were aggregated to both leukocyte subpopulations, no significant differences between active or not active smokers COPD patients were observed, but a tendency for enhanced CXCR6 expression was noted in the former group (Figures 5C–F).

### Circulating Leukocytes from Patients with COPD Have Greater Adhesiveness to CSE-Stimulated HUAEC

**Figure 6:**
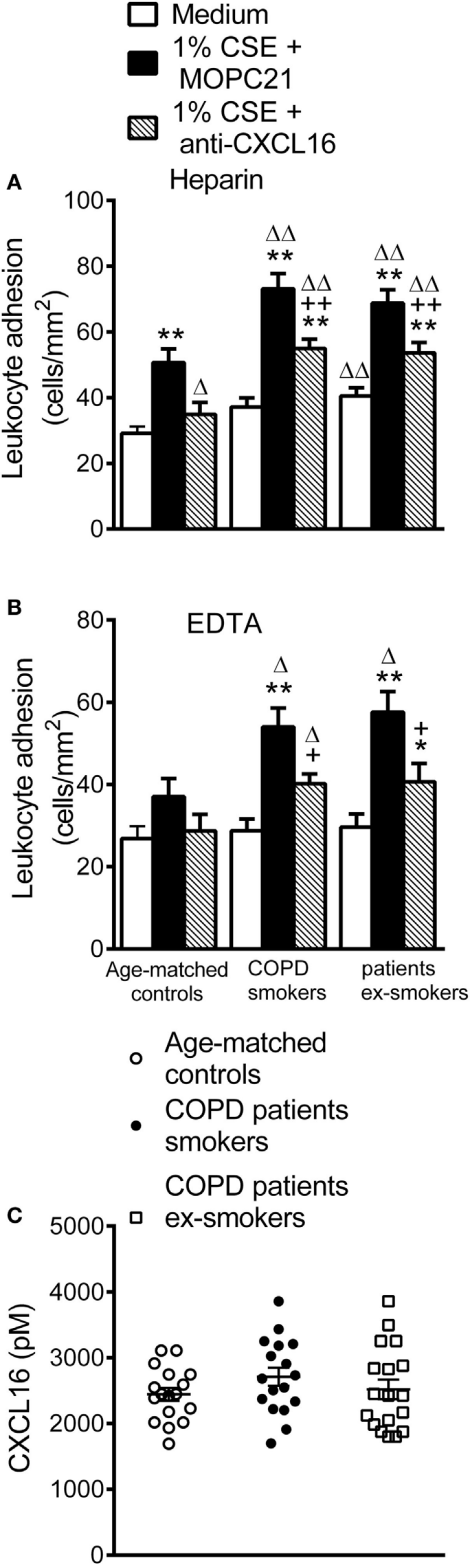
Leukocyte recruitment by cigarette smoke extract (CSE)-stimulated human umbilical arterial endothelial cells (HUAEC) and CXCL16 plasma levels from whole blood of active and not active smokers patients with chronic obstructive pulmonary disease (COPD) and aged-matched controls. HUAEC were stimulated with 1% CSE for 24 h. Some cells were incubated with a CXCL16 neutralizing antibody (2 µg/ml) or an irrelevant isotype-matched monoclonal antibody (MOPC-21, 2 µg/ml). Subsequently, whole blood from patients with COPD active or not active smokers and healthy aged-matched controls incubated **(A)** without, or **(B)** with EDTA, was perfused over endothelial monolayers for 5 min at 0.5 dyn/cm^2^ and leukocyte adhesion quantified (*n* = 13 aged-matched controls, *n* = 14 active smokers COPD patients, *n* = 16 ex-smokers COPD patients). Values are expressed as the mean ± SEM. **P < 0.01 relative to values in the medium group; ^+^*P* < 0.05 or ^++^*P* < 0.01 relative to 1% CSE group; ^Δ^*P* < 0.05 or ^ΔΔ^*P* < 0.01 relative to the values in the aged-matched control group. **(C)** CXCL16 plasmatic levels were measured by ELISA (*n* = 17 aged-matched controls, *n* = 17 active smokers COPD patients, *n* = 18 ex-smokers COPD patients). Values are expressed as the mean ± SEM.

To explore the functional consequences of these observations, we examined the involvement of platelet CXCR6 on CXCL16-dependent leukocyte–endothelial cell interactions under dynamic conditions. When heparinized whole blood from COPD patients and aged-matched controls was perfused across unstimulated HUAEC, leukocyte adhesiveness was higher in the COPD group (Figure 6A). No differences were found between active and ex-smokers COPD patients (Figure 6A). Leukocyte adhesiveness was significantly greater after exposure of HUAEC to 1% CSE (Figure 6A). Interestingly, neutralization of CXCL16 activity on endothelial cells resulted in a significant reduction of CSE-induced platelet–leukocyte–endothelial adhesion in the three groups (Figure 6A).

Importantly, when platelets were disaggregated from leukocytes, cells from control subjects did not adhere significantly better to CSE-stimulated endothelium than to unstimulated (medium only) HUAEC (Figure 6B). By contrast, a significant increase in leukocyte adhesion to the CSE-stimulated endothelium was observed in the COPD group irrespective of smoking activity (Figure 6B), and neutralization of CXCL16 activity again markedly decreased CSE-induced leukocyte adhesion in the COPD group (Figure 6B). However, no significant differences in the circulating levels of soluble CXCL16 were found between the groups (Figure 6C).

### CS-Induced Leukocyte Adhesion to Mouse Cremasteric Arterioles Is Reduced in CXCR6^−/−^ Mice

**Figure 7:**
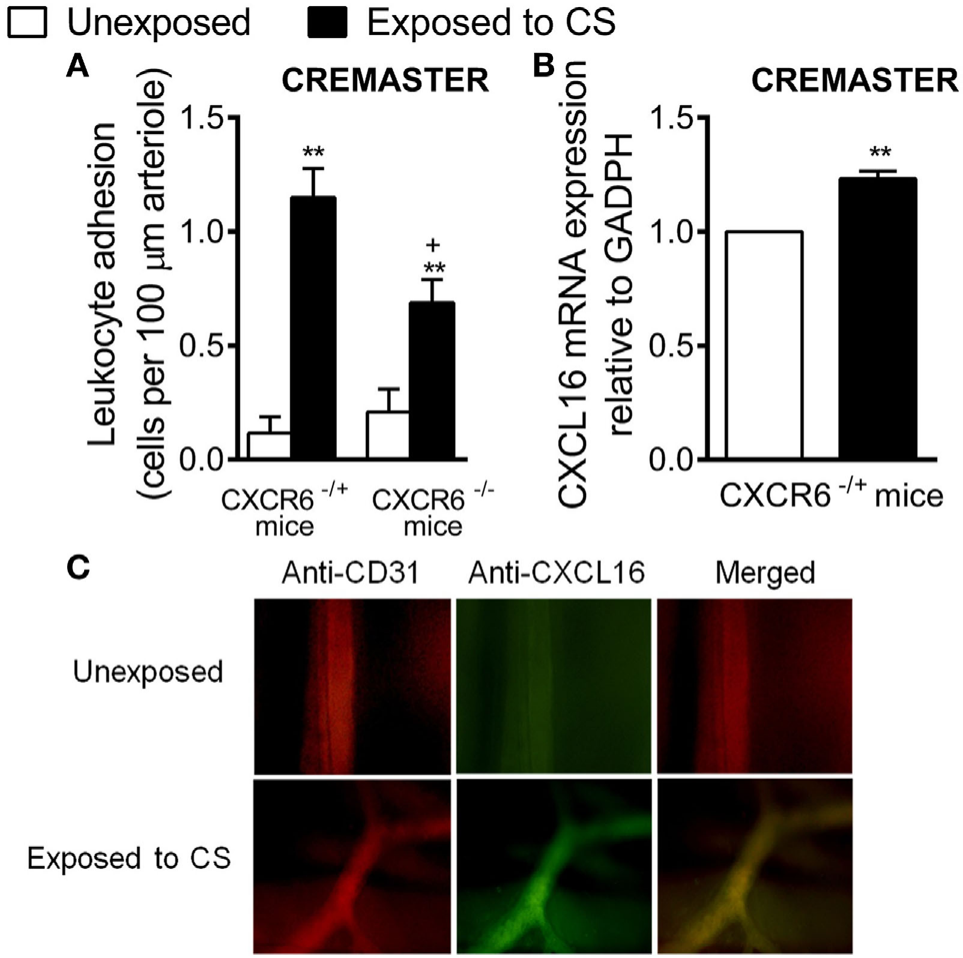
Effect of cigarette smoke (CS) exposure in CXCR6-expressing and CXCRC6 knockout mice. Heterozygous (CXCR6^−/+^) and homozygous (CXCR6_−/–_) mice were exposed or not to CS for 3 days and responses were examined 16 h later. **(A)** Leukocyte–arteriolar endothelium interactions was measured by intravital microscopy. Results are expressed as mean ± SEM (*n* = 5–8 animals per group). *P < 0.05 or **P < 0.01 relative to non-exposed animals; ^+^*P* < 0.05 relative to CXCR6^−/+^ mice. **(B)** Relative quantification of CXCL16 and β-actin mRNA was determined by RT-PCR. Columns show fold increase in expression of CXCL16 mRNA relative to control GAPDH values (*n* = 5 independent experiments). Values are represented as mean ± SEM of the 2^−ΔΔCt^ values. **P < 0.01 relative to non-exposed animals. **(C)** Cremaster muscle was fixed for CXCL16 and endothelium (CD31) staining. CXCL16 expression is shown in green (stained with an Alexa Fluor 488-conjugated donkey anti-rabbit secondary antibody) and vessel endothelium (red) was stained with a PE-conjugated anti-mouse CD31 monoclonal antibody. Overlapping expression of CXCL16 and CD31 is shown in yellow. Results are representative of five to six animals per group.

To explore the potential *in vivo* relevance of these findings, we used a murine model of acute CS exposure in a background of CXCR6 deficiency. While CS exposure induced a significant increase in arteriolar leukocyte adhesion in the cremaster muscle in both strains as assessed by intravital microscopy, leukocyte adhesion was significantly attenuated in CXCR6^−/−^ mice (39% inhibition, Figure 7A). Finally, mRNA and immunohistochemistry analysis of the cremasteric microcirculation demonstrated increased expression of CXCL16 in CS-exposed animals (Figures 7B,C).

## Discussion

Cardiovascular diseases are common comorbidities in smokers and patients with COPD (3); however, the mechanisms by which they develop remain largely unknown. Our study provides the first demonstration that water-soluble components of CS induce arterial endothelial dysfunction partly through functional enhanced endothelial CXCL16 expression. This effect was dependent on Nox5 expression and on subsequent RhoA and p38 MAPK activation, leading to NF-κB transactivation and presumably further upregulation of CXCL16 gene expression. Peripheral blood analysis of patients with COPD revealed greater numbers of activated circulating platelets (PAC-1^+^ and P-selectin^+^) and enhanced levels of circulating CXCL16- and CXCR6-expressing platelets. This activation was associated with augmented platelet–leukocyte adhesion to CSE-stimulated human arterial endothelium, which was partially dependent on arterial CXCL16 upregulation and increased CXCR6 expression on platelet–leukocyte aggregates and mononuclear cells. Furthermore, acute CS exposure *in vivo* was accompanied by increased CXCL16 expression in murine cremasteric arterioles, an organ distant from the lung, and leukocyte–arteriolar adhesion was significantly impaired in animals with a nonfunctional CXCR6 receptor. Overall, these results suggest that increased CXCR6 expression on mononuclear leukocytes and platelet–leukocyte aggregates might constitute a biomarker of systemic inflammation, which seems to be the main driver of CVD development, since CXCR6 is involved in the enhanced leukocyte adhesion to the dysfunctional arterial endothelium of COPD patients.

The use of aqueous CSE for cell stimulation is the most widely employed *in vitro* model for studies of COPD development and CS impact on cardiovascular outcomes (32–36). Despite its common use, it brings with it some limitations given that CS contains a large number of components (35), and there is a lack of knowledge of their identity and concentrations in smokers’ circulation (7). Nevertheless, aqueous CSE has a comparatively constant chemical composition (34). Indeed, the hydrophilic fraction of CS is believed to mainly contain the atherosclerosis-causing agents (34). Accordingly, direct and indirect effects of CS on leukocyte–endothelial cell adhesion have been reported (37); directly, through oxidants and free radicals generated by CS, and indirectly, through the generation of pro-inflammatory cytokines (37). Of note, cigarette use induces endothelial dysfunction and increased circulating levels of inflammatory cytokines such as TNF-α, a cytokine which has been detected in the circulation of smokers and COPD patients (3, 7, 32, 35). We show here that CSE stimulation or acute exposure of mice to CS increases CXCL16 expression on arterial endothelial cells and cremasteric arterioles and enhances CXCL16-mediated mononuclear cell adhesiveness. It, therefore, seems plausible that this effect will be the consequence of either a direct effect of the water-soluble components of CS on endothelial cells, or the generation and release of TNF-α from the stimulated endothelium. Indeed, we have previously found that CSE-induced mononuclear recruitment was abolished in TNF-α-silenced endothelial cells (18), and TNF-α is one of the key cytokines involved in endothelial CXCL16 upregulation (11).

By exploring the mechanisms involved in CSE-induced CXCL16 endothelial expression, we found that Nox5 silencing decreased chemokine expression. Indeed, CS exposure induces rapid ROS production, impairing endothelial functions (38). Mononuclear cell recruitment induced by CS was also found to be Nox5 dependent (18). Additionally, the RhoA/Rho kinase pathway has been implicated in leukocyte recruitment (39) and RhoA can be activated by Nox5-generated ROS (40). We show that pharmacological inhibition of RhoA or its knockdown in HUAEC diminished CXCL16 expression provoked by CSE. RhoA is an upstream regulator of MAPK family members such as p38 MAPK (41), which can regulate the transcription of many genes through its action on downstream targets such as NF-κB; both are involved in inflammatory responses such as mononuclear cell recruitment induced by CSE (18). Our findings suggest that CSE-induced CXCL16 arterial upregulation is a consequence of RhoA activation by oxidant generation and p38 MAPK. This leads to activation of NF-κB and the further regulation of genes including CXCL16, which actively participates in the mononuclear leukocyte recruitment induced by CSE.

To extend our findings to a clinically relevant scenario, we studied different parameters in three homogeneous groups: control subjects with normal lung function and smokers and ex-smokers COPD patients. We first investigated platelet activation and expression of CXCL16 and CXCR6 in these groups. Platelet activation is linked to cardiovascular morbidity; indeed, activated platelets can mediate the endothelial adhesion of circulating leukocytes (6). COPD has been associated with platelet activation (42). Our present results confirm and extend these findings and show that patients with COPD have significantly more platelets expressing CXCL16 and CXCR6 than do age-matched controls, while no significant differences were found between smokers and ex-smokers COPD patients, there was a tendency for an increased number in the former. Because CXCR6 is highly expressed in platelets (43), we analyzed its expression on different platelet-bound or unbound leukocyte subsets. Surprisingly, we found higher circulating levels of CXCR6^+^-platelets bound to neutrophils in COPD patients than in age-matched control subjects. Typically, this subset of leukocytes does not express CXCR6 (11), which was confirmed by the absence of expression of the CXCL16 receptor when platelets were dissociated (EDTA). In parallel, increased circulating numbers of CXCR6^+^ platelet–monocyte/lymphocyte aggregates and CXCR6^+^ mononuclear cells were found in COPD patients, although again no significant differences were observed between the smoker and ex-smoker groups.

These findings have functional consequences and we show that platelet–leukocyte adhesion to CSE-stimulated HUAEC was significantly more potent in the COPD group irrespective of their smoking activity. Our findings also suggest that platelets are critical for leukocyte adhesion to CSE-stimulated dysfunctional arterial endothelium, as in their absence no significant adhesion was found in aged-matched controls. In this context, platelets induce leukocyte recruitment in multiple inflammatory disorders, a property dissociated from their role in hemostasis (44). Therefore, in aged-matched controls, platelet-bound neutrophils are likely responsible for the arterial interactions detected since CSE-induced increased adhesiveness was partly dependent on CXCL16 upregulation and neutrophils do not express the CXCL16 receptor (CXCR6) (11), but platelets do (43). Notably, in the setting of COPD, platelet–leukocyte aggregates or platelet-unbound leukocytes showed similar adhesion to unstimulated and stimulated arterial endothelium. Moreover, CXCL16 neutralization exerted a comparable and significant reduction of CSE-induced leukocyte adhesion, both in untreated and in EDTA-treated whole blood. This parity can be attributed to the low shear rate employed, wherein platelet–leukocyte or leukocyte adhesion is platelet independent as described previously (45). Despite these findings, no differences in the levels of circulating soluble CXCL16 were found between groups. Finally, the clinical impact of these findings is likely relevant. First, patients with metabolic syndrome, who are also prone to atherosclerosis development, have increased numbers of circulating CXCR6^+^ cells (46). Second, blockade of CXCR6/CXCL16 axis reduces both platelet and leukocyte attachment to the arterial endothelium in COPD. It is, therefore, attainable that increased numbers of circulating CXCR6-expressing platelets and mononuclear cells may establish a direct link between COPD and endothelial dysfunction and the further development of cardiovascular disorders. Nevertheless, no significant differences in the parameters studied were encountered between COPD smokers and ex-smokers, suggesting that once the disease is established both populations are at similar risk of developing CVD.

In conclusion, we provide evidence that CSE induces CXCL16-dependent leukocyte arrest by the arterial endothelium through Nox5 expression and RhoA/p38 MAPK/NF-κB activation. Increased numbers of circulating CXCR6-expressing platelets and mononuclear cells of COPD patients may constitute a marker of systemic inflammation with potential consequences in CVD development. Moreover, our study provides new insights into the potential therapeutic use of CXCL16/CXCR6 axis blockade since it dramatically reduces the adherence of platelets and leukocytes from COPD patients to the dysfunctional arterial endothelium, and treatment of cardiovascular comorbidities is a key goal in the management of this patient group.

## Ethics Statement

All investigation with human samples conformed to the principles outlined in the Declaration of Helsinki and was approved by the institutional ethics committee at the University Clinic Hospital of Valencia. Written informed consent was obtained from all subjects. The animal protocol conforms to the Guide for the Care and Use of Laboratory Animals published by the US National Institutes of Health (NIH publication No. 85-23, revised 1996) and was approved by the Ethics Review Board of the University of Valencia.

## Author Contributions

M-JS, LP, CG, and ES designed the study and PM, AC, LP, CG, ES, and M-JS wrote the paper. All authors performed experiments, analyzed and interpreted the data. All authors revised the work and approved the version to be published.

## Conflict of Interest Statement

The authors declare that the research was conducted in the absence of any commercial or financial relationships that could be construed as a potential conflict of interest.
