# Supplementary material for: Cigarette Smoke Increases Endothelial CXCL16-Leukocyte CXCR6 Adhesion In Vitro and In Vivo. Potential Consequences in Chronic Obstructive Pulmonary Disease
Source: Front Immunol. 2017 Dec 13;8:1766. doi: 10.3389/fimmu.2017.01766 (PMC5733535; doi:10.3389/fimmu.2017.01766)
Supplement: Supplementary file 1 [file Data_Sheet_1.doc]

**Supplemental Material**

**Cigarette smoke increases endothelial CXCL16-leukocyte CXCR6 adhesion *in vitro* and *in vivo*. Potential consequences in COPD.**

^1,2^Patrice Marques^#^, ^1,2^Aida Collado^#^,  ^1,2^Paula Escudero, ^1,2^Cristina Rius, ^2,3^Cruz González, ^2,3^Emilio Servera,  ^2^Laura Piqueras*, ^1,2^Maria-Jesus Sanz*.

**
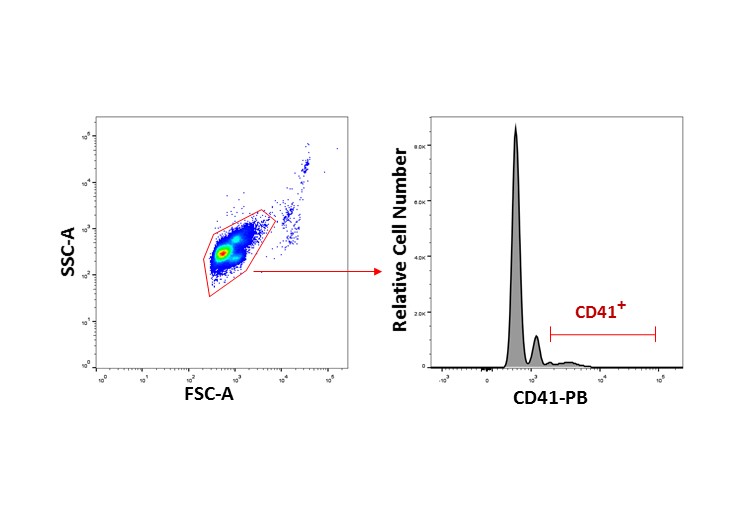
Figure I: Flow cytometry detection and morphologic gating of human platelets in whole blood.** Platelets were gated according to the side scatter in the logarithmic scale and defined as CD41^+^ population.

**
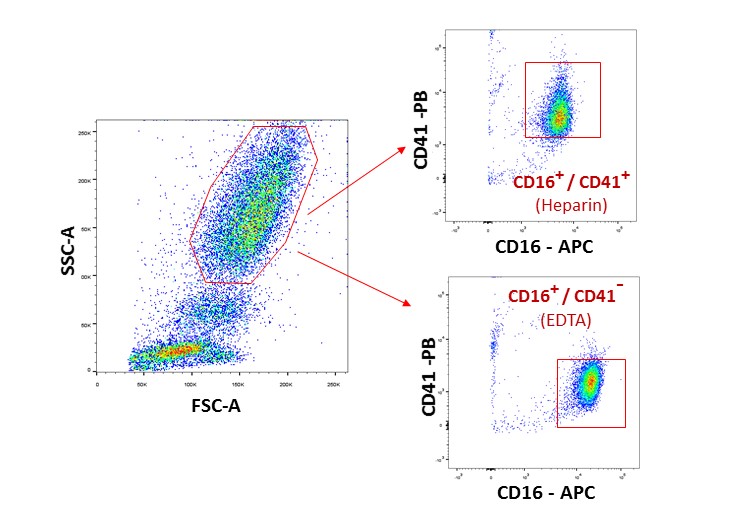
Figure II: Flow cytometry detection and morphologic gating of human neutrophils in whole blood**. Neutrophils-platelets complexes were selected as CD16^+^ CD14^-^ CD41^+^ population from heparinized whole blood, and platelet-free neutrophils were gated as CD16^+^CD14^-^CD41^-^ from blood incubated with EDTA.

**
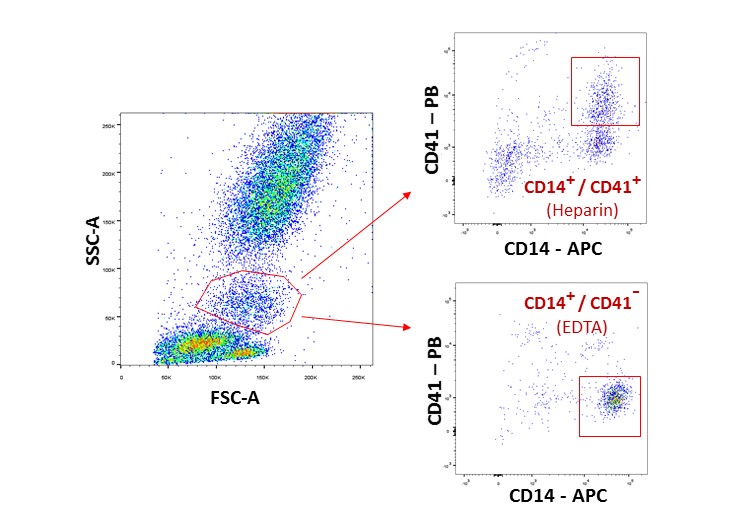
Figure III: Flow cytometry detection and morphologic gating of human monocytes in whole blood**: Monocytes-platelets complexes were selected as CD14^+^CD41^+^ population from heparinized whole blood, and platelet-free monocytes were gated as CD14^+^CD41^-^ from blood incubated with EDTA.

**
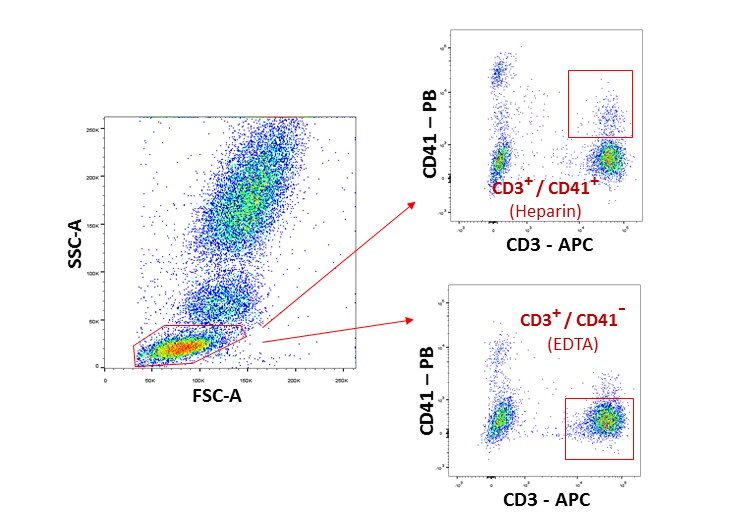
Figure IV: Flow cytometry detection and morphologic gating of human lymphocytes in whole blood**. Lymphocytes-platelets complexes were selected as CD3^+^ CD41^+^ population from heparinized whole blood, and platelet-free lymphocytes were gated as CD3^+^CD41^-^ from blood incubated with EDTA.

**Table I**: Patient demographics of the subjects studied (data expressed as mean ± SEM)

|  | **Control**  **non-smoker volunteers** | **Smokers COPD Subjects** | **Ex-smokers COPD Subjects** |
| --- | --- | --- | --- |
| **Numbers per group (n)** | 17 | 17 | 18 |
| **Smoking (Pack-year)** | NA | 50.4 ± 4.0 | 50.7 ± 5.0 |
| **Age (years)** | 65.1 ± 2.2 | 62.2 ± 2.0 | 68.1 ± 1.5 |
| **FEV1 (% Predicted)** | 103.2 ± 4.6 | 57.2 ± 4.2** | 47.0 ± 3.8** |
| **FEV 1/FVC (%)** | 79.9 ± 1.7 | 53.7 ± 2.8** | 48.3 ± 1.7** |
| **Gender (M)** | 100 | 88.2% | 94.4% |

*Definition of abbreviations*: Pack-year (nº cigarettes per day per smoking years / 20); NA = not applicable; FEV 1 % Predicted, forced expiratory volume in 1 s (%); FVC, forced vital capacity. ***P* < 0.01 versus control group.

**Table II**: Control subjects and COPD patient comorbidities

|  | **Control**  **non-smoker volunteers** | **Smokers COPD Subjects** | **Ex-smokers COPD Subjects** |
| --- | --- | --- | --- |
| **Hypertension** | 41% | 40% | 39% |
| **Dyslipidemia** | 29% | 13% | 22% |
| **Type 2 Diabetes** | 18% | 20% | 17% |
| **Obesity** | 6% | 0% | 6% |





**Figure V*.*** Effect of CXCL16 neutralizing antibody on TNF-α-induced neutrophil and mononuclear leukocyte adhesion on HUAEC. Endothelial cells were stimulated with TNF-α (20 ng/ml) for 24 h. Some cells were incubated with a CXCL16 neutralizing antibody (2 μg/ml) or an irrelevant isotype-matched monoclonal antibody (MOP-C21, 2 μg/ml). Subsequently, human neutrophils (A) or mononuclear cells (B) (1×10^6^ cells/ml) incubated with or without EDTA, were perfused over the monolayers for 5 min at 0.5 dyn/cm^2^ and leukocyte accumulation quantified (n=4 independent experiments). Values are expressed as the mean ± SEM. ***P* < 0.01 relative to values in the medium group; ^+^*P* < 0.05 relative to the stimulus MOPC-21-treated group.

**
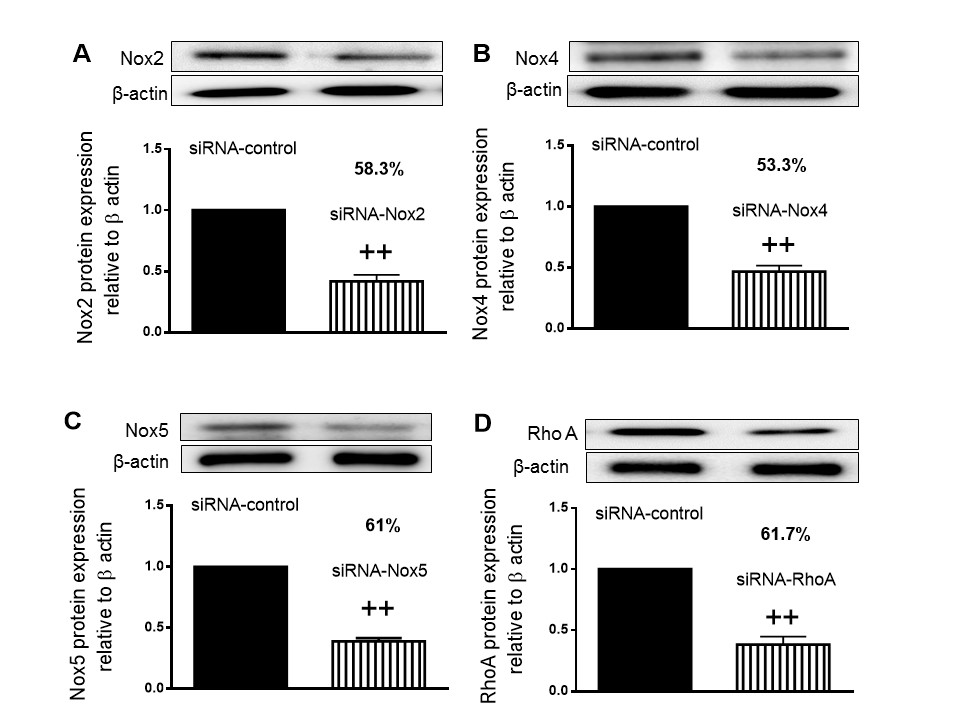
Figure VI: Transfection of HUAEC with Nox 2, Nox 4, Nox 5 or RhoA specific siRNAs.** Gene silencing was performed in HUAEC using either control siRNA or specific siRNA to Nox2, Nox4, Nox5 or RhoA. After 48 h, protein expression of Nox2 (A), Nox4 (B), Nox5 (C) or RhoA (D) was determined by western blot to assess silencing efficiency. Results (mean ± SEM of n=4–5 independent experiments) are expressed relative to β-actin. Representative blots are shown. ^++^*P* < 0.01 relative to values in the respective control siRNA group.
